# SARS-CoV-2 infection, neuropathogenesis and transmission among deer mice: Implications for spillback to New World rodents

**DOI:** 10.1371/journal.ppat.1009585

**Published:** 2021-05-19

**Authors:** Anna Fagre, Juliette Lewis, Miles Eckley, Shijun Zhan, Savannah M. Rocha, Nicole R. Sexton, Bradly Burke, Brian Geiss, Olve Peersen, Todd Bass, Rebekah Kading, Joel Rovnak, Gregory D. Ebel, Ronald B. Tjalkens, Tawfik Aboellail, Tony Schountz

**Affiliations:** 1 Department of Microbiology, Immunology and Pathology, College of Veterinary Medicine, Colorado State University, Fort Collins, Colorado, United States of America; 2 Department of Environmental and Radiological Health Sciences, College of Veterinary Medicine, Colorado State University, Fort Collins, Colorado, United States of America; 3 Department of Biochemistry and Molecular Biology, College of Natural Sciences, Colorado State University, Fort Collins, Colorado, United States of America; 4 Veterinary Diagnostic Laboratory, College of Veterinary Medicine and Biomedical Sciences, Colorado State University, Fort Collins, Colorado, United States of America; Washington University in Saint Louis School of Medicine, UNITED STATES

## Abstract

Coronavirus disease-19 (COVID-19) emerged in late 2019 in China and rapidly became pandemic. As with other coronaviruses, a preponderance of evidence suggests the virus originated in horseshoe bats (*Rhinolophus* spp.) and may have infected an intermediate host prior to spillover into humans. A significant concern is that SARS-CoV-2 could become established in secondary reservoir hosts outside of Asia. To assess this potential, we challenged deer mice (*Peromyscus maniculatus*) with SARS-CoV-2 and found robust virus replication in the upper respiratory tract, lungs and intestines, with detectable viral RNA for up to 21 days in oral swabs and 6 days in lungs. Virus entry into the brain also occurred, likely via gustatory-olfactory-trigeminal pathway with eventual compromise to the blood-brain barrier. Despite this, no conspicuous signs of disease were observed, and no deer mice succumbed to infection. Expression of several innate immune response genes were elevated in the lungs, including IFNα, IFNβ, Cxcl10, Oas2, Tbk1 and Pycard. Elevated CD4 and CD8β expression in the lungs was concomitant with Tbx21, IFNγ and IL-21 expression, suggesting a type I inflammatory immune response. Contact transmission occurred from infected to naive deer mice through two passages, showing sustained natural transmission and localization into the olfactory bulb, recapitulating human neuropathology. In the second deer mouse passage, an insertion of 4 amino acids occurred to fixation in the N-terminal domain of the spike protein that is predicted to form a solvent-accessible loop. Subsequent examination of the source virus from BEI Resources determined the mutation was present at very low levels, demonstrating potent purifying selection for the insert during in vivo passage. Collectively, this work has determined that deer mice are a suitable animal model for the study of SARS-CoV-2 respiratory disease and neuropathogenesis, and that they have the potential to serve as secondary reservoir hosts in North America.

## Author summary

A significant concern is that SARS-CoV-2 could establish in natural wildlife populations that could lead to transmission events to humans. We have determined that deer mice are susceptible to SARS-CoV-2 and that virus can persist for up to 21 days. Moreover, efficient transmission to other deer mice occurred, suggesting the potential for sustained persistence in natural populations of deer mice. The pathology observed in the respiratory tract resembles that which occurs in human COVID-19 patients, including robust inflammation and infiltration of neutrophils and macrophages, and neurological manifestations in the olfactory bulb and tongue that could impact senses of smell and taste, respectively. Collectively, the work suggests deer mice could serve as secondary reservoir hosts of SARS-CoV-2 and as an animal model for COVID-19 disease studies.

## Introduction

Coronavirus disease-19 (COVID-19) emerged in late 2019 in China and the etiologic agent was identified as a novel betacoronavirus, severe acute respiratory syndrome coronavirus-2 (SARS-CoV-2) [1–3]. Both SARS-CoV and SARS-CoV-2 use angiotensin converting enzyme 2 (ACE2) as a cellular entry receptor in humans [2,4]. The virus likely originated from insectivorous *Rhinolophus* spp. horseshoe bats. Some evidence suggests it may have undergone recombination in the receptor binding domain via an intermediate host prior to its spillover into humans [2,5]. However, other evidence suggests the virus has not undergone recombination in its receptor binding domain [6] and may have transmitted to humans directly from bats or via another intermediate host. To date, only a few mammalian species have been identified as susceptible, including cynomolgus macaques, ferrets, felines, American mink, Egyptian rousette bats and canines [7–13]. Human ACE2-transgenic mice are also susceptible, unlike wildtype laboratory mice [14].

A significant concern is the potential for spillback of SARS-CoV-2 into native wildlife species that could allow the virus to become endemic by the establishment of secondary reservoir hosts outside of Asia [15]. Middle East respiratory syndrome coronavirus (MERS-CoV) likely transmitted from bats to dromedary camels in North Africa and established a secondary reservoir that accounts for most human outbreaks of MERS which repeatedly occur each year [16,17]. A recent report identified 20 important contact residues in human ACE2 for SARS-CoV-2 spike protein binding and suggested members the rodent Cricetidae family may also be susceptible [18]. Experimental challenge of Syrian hamsters, a cricetid rodent whose ACE2 shares 18 of these 20 critical residues, resulted in moderate, nonlethal pulmonary disease resembling human COVID-19 but without mortality [7,8,13].

Peromyscine rodents are also members of Cricetidae (subfamily Neotominae, genus *Peromyscus*) and are distributed throughout North America. There are 56 recognized species in the genus, and some serve as reservoir hosts for diverse zoonotic agents, including hantaviruses, *Borrelia burgdorferi*, *Babesia* spp., and Powassan virus [19]. Deer mice (*P*. *maniculatus*) are the most studied and abundant mammals in North America and are frequently contacted by mammalogists during field studies [20]. They are also abundant in regions where American mink (*Neovison vison*) are farmed, raising the possibility of contact with infected American mink or fomites (e.g., mink food) that may be contaminated with SARS-CoV-2. The ACE2 receptor of deer mice shares 17 of the 20 critical residues for SARS-CoV-2 binding (S1 Table), suggesting that deer mice may be susceptible to infection with SARS-CoV-2. Because of these features, we challenged deer mice to determine if they are susceptible and whether they can transmit the virus through multiple passages.

## Results

### Susceptibility testing

**Fig 1:**
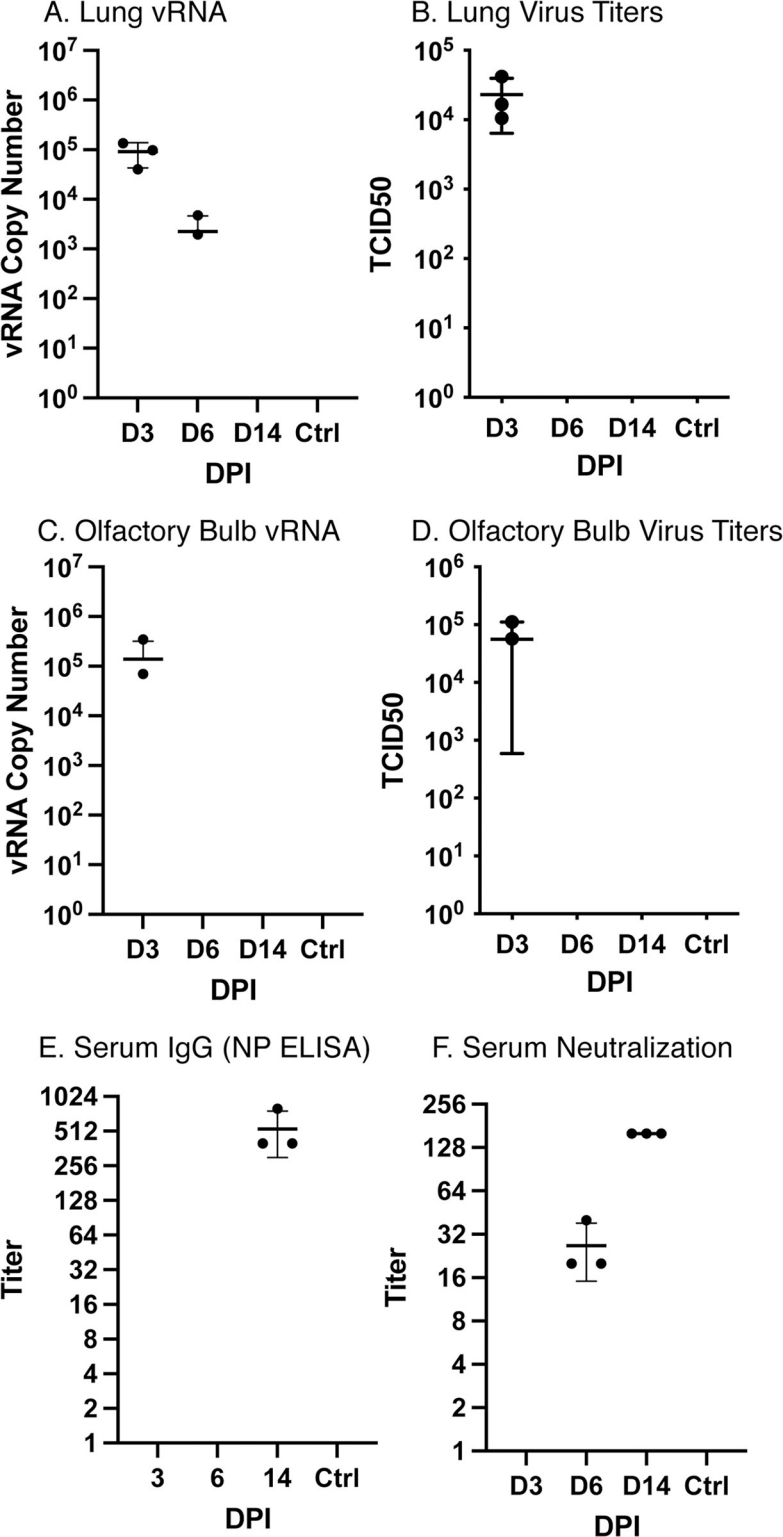
Infection and antibody response to SARS-CoV-2. (A) Viral RNA was detected in lungs of infected deer mice to day 6, and (B) virus was recovered only on day 3. (C) Two of three deer mice had detectable vRNA in their olfactory bulbs and (D) virus was isolated from each. (E) IgG antibodies were detected to nucleocapsid protein on day 14, (F) with neutralizing antibody detected on days 6 and 14. Each time point represents samples collected from 3 euthanized deer mice per group. Error bars represent the standard deviation of the mean and geometric mean antibody titers.

To test susceptibility, nine young adult deer mice 6 months of age of both sexes were intranasally challenged with 2x10^4^ TCID_50_ of SARS-CoV-2 and three were held as unchallenged controls. Three deer mice each were euthanized on days 3, 6 and 14 post inoculation (dpi) to assess infection, and one sham-inoculated deer mouse was euthanized on day 3 whereas the other two were euthanized on day 14. On days 3 and 6, gross substantial pulmonary consolidation and hemorrhage were observed in the cranial and middle portions of both lungs. Viral RNA was detected in the lungs of all deer mice euthanized on days 3, and two of three on day 6, but virus was isolated from only the day 3 deer mice (Fig 1A and 1B). Virus and viral RNA was detected in olfactory bulbs of two deer mice on day 3 but not on subsequent days (Fig 1C and 1D). IgG to recombinant nucleoprotein was detected by ELISA only on day 14 (Fig 1E; GMT = 504), and low titer neutralizing antibody (GMT = 25) was initially detected on day 6 that significantly increased on day 14 (GMT = 160, Fig 1F), suggesting neutralizing antibody on day 6 was likely IgM that prevented virus isolation in the day 6 deer mice. Antibody to multiple viral antigens was detected in all 9 of the inoculated deer mice by western blot (S1 Fig), indicating an early antibody response.

### Immune gene expression

**Fig 2:**
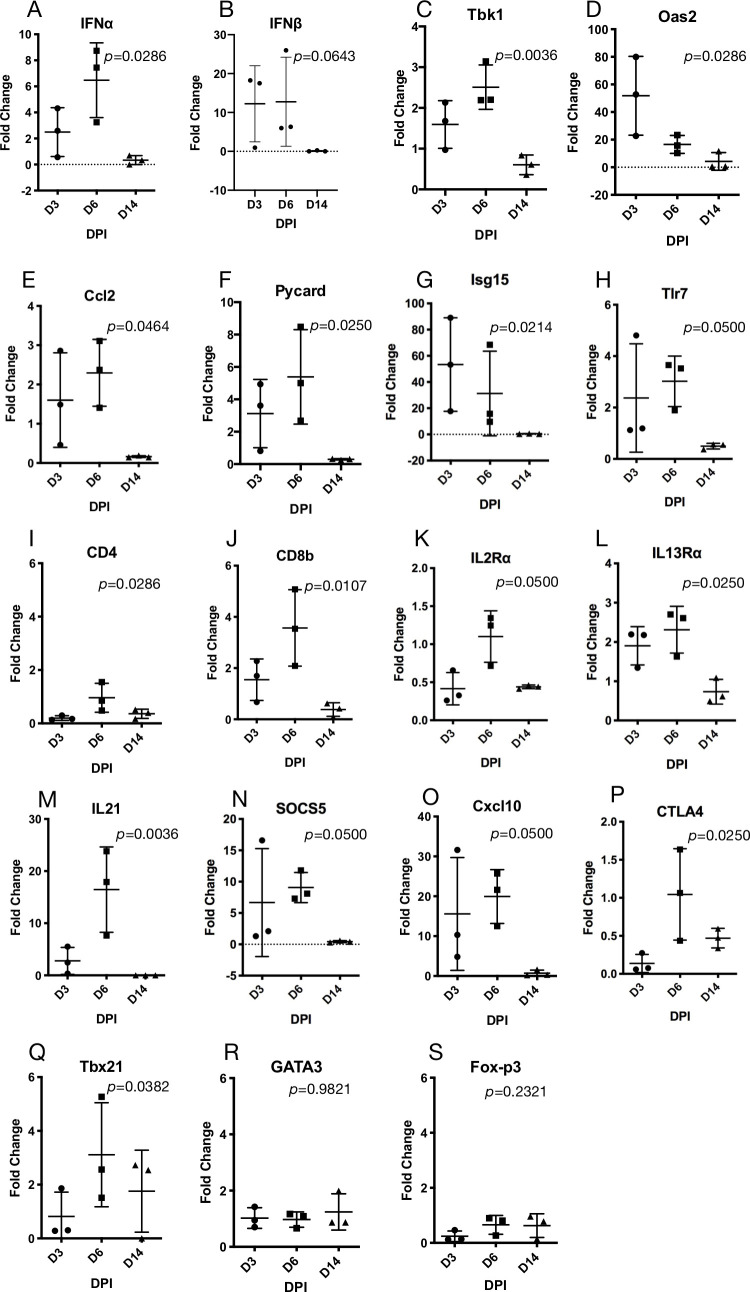
Immune gene expression in lungs of infected deer mice. Gene profiling showed elevated expression of several antiviral innate immune response genes 3 and 6 dpi (A-H) and evidence of T cell infiltration and inflammatory type I immune response 6 dpi (I-S).

Examination of 41 immune response genes [21] (S2 Table) identified several that were elevated in the lungs during infection. Seven innate immune response genes (IFNα, Tbk1, Oas2, Cxcl10, Pycard, Isg15, Tlr7) were significantly elevated during acute infection but then subsided by 14 dpi; IFNβ expression was elevated but not significantly so (Fig 2A–2H), indicating activation of antiviral defenses that declined as the virus was controlled. IL-6 expression was not elevated; high levels of IFN and IL-6 have been associated with severe COVID-19 [22]. Expression of CD8β was substantially elevated on days 3 and 6, whereas CD4 expression was less elevated; both returned to nominal levels by day 14 (Fig 2I–2J). IFNγ expression was not detected in the lungs of the sham inoculated deer mice; however, it was detected at low levels in one deer mouse at 3 dpi (Cq = 35) and two deer mice at 6 dpi (Cq = 32, 35). Although IL-2 and IL-13 were not detected, receptors for each were significantly elevated (Fig 2K–2L). IL-21 expression was greatest among the cytokines examined with up to 25-fold increase 6 dpi (Fig 2M). SOCS5, Cxcl10 and CTLA4 were each significantly elevated on day 6 (Fig 2N–2P). The type I inflammatory immune response transcription factor Tbx21 (T-bet) was significantly elevated, but not the type II GATA3 or regulatory T cell Foxp3 transcription factors (Fig 2Q–2S), suggestive of a pro-inflammatory type I immune response in the lungs of infected deer mice.

### Histopathology

**Fig 3:**
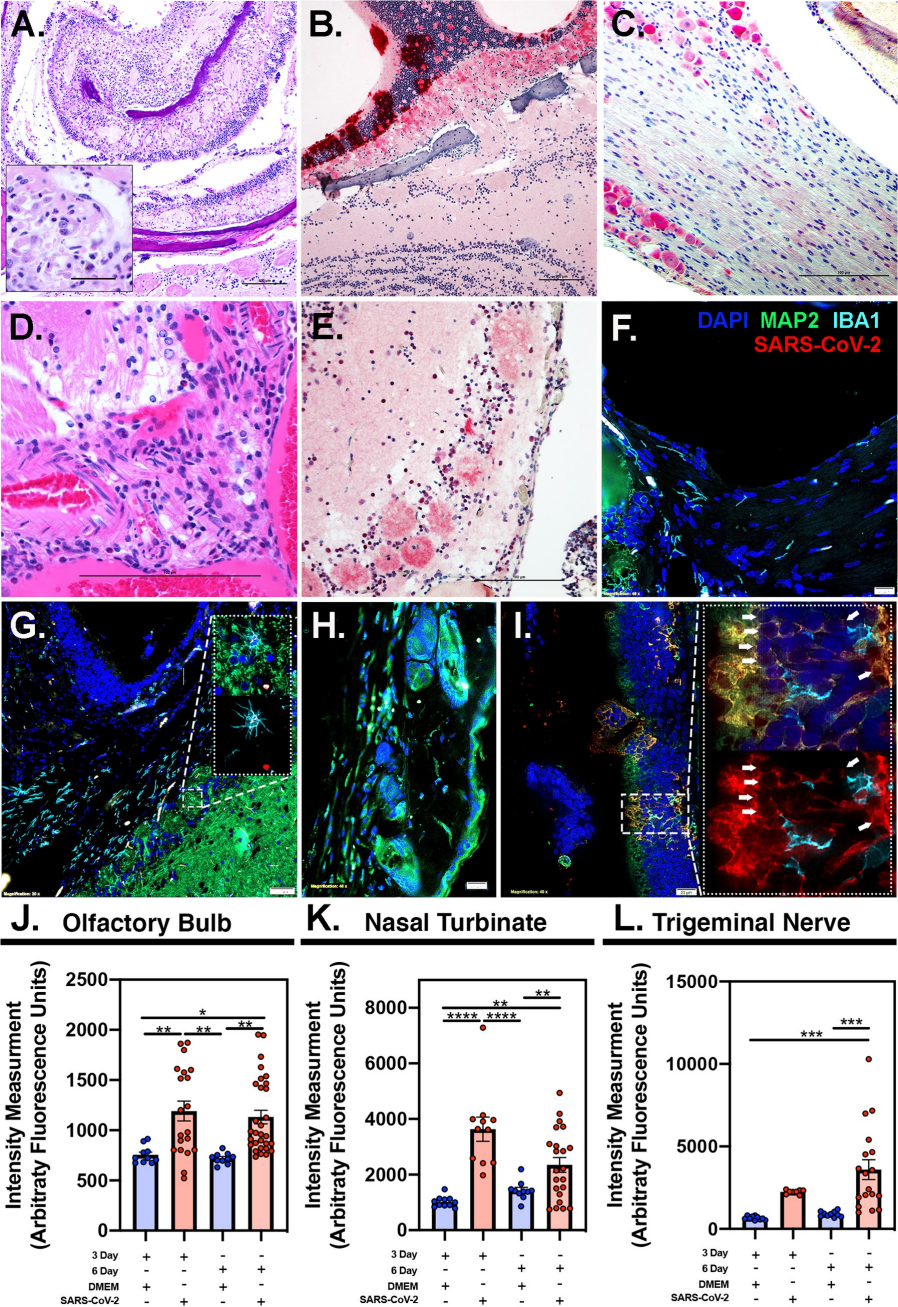
Histopathology and immunohistochemistry of SARS-CoV-2 in skull and brain of deer mice at 3- and 6- days post-infection. (A) Acute fibrinosuppurative and ulcerative sinusitis in ethmoturbinates with degeneration and inflammation of branches of olfactory, ethmoidal, and maxillary sensory nerves (fibrinoid vascular necrosis–inset) (B) Transmural SARS-CoV-2 immunoreactivity (Fast Red staining) in MOE, 3 dpi. (C) Prominent immunoreactivity in trigeminal ganglionic neurons with mild glial reaction at 6 dpi. (D) Disruption of the BBB with associated histioneutrophilic meningoencephalitis, 6 dpi (E) Viral transmission to the glomerular layer of the MOB, 6 dpi. (F) Immunofluorescence imaging depicting entry of centrifugal afferents to the olfactory bulb at the cribriform plate in uninfected control deer mice 6 dpi showing no viral immunoreactivity in any neurons or glial cells. Neurons were identified with antibodies against microtubule associated protein (MAP2, green), microglia with anti-ionized calcium-binding adaptor molecule 1 (IBA1, cyan), anti-SARS-CoV-2 (red) and nuclei were counterstained with DAPI (blue). (G) Multifocal SARS-CoV-2 antigen was detected by 6 dpi within neurons and microglia of the afferent nerves and in the glomerular layer of the olfactory bulb (arrows; 100X high-magnification inset microglial cell). Examination of the trigeminal nerve and ganglion in (H) uninfected control with no immunoreactivity and (I) infected deer mice revealing neuroinvasion of SARS-CoV-2, with extensive co-localization of the virus within MAP2^+^ neurons proximal to activated microglia, 6 dpi. 100X high-magnification insets depict co-localization of SARS-CoV-2 with MAP2^+^ neurons (top panel, arrows), as well as the same image without MAP2 to better highlight the extent of neuronal staining with SARS-CoV-2. SARS-CoV-2 quantification by fluorescence intensity within given regions of interest (ROI) are represented in the olfactory bulb (J), nasal turbinates (K), and trigeminal nerve (L) showing the increase in viral load. Scale bars equal 100 μm (n = 2/group) *p<0.0332, **p<0.0021, ***p<0.0002, ****p<0.0001.

Histologically, at 3 dpi, multifocal immunoreactivity was seen in occasional vibrissae with no associated inflammation in mystacial pad. Hemi skulls showed severe necrosuppurative to fibrinopurulent inflammation in nasal meatuses, maxillary sinuses and ethmoturbinates. Fibrinoid vascular necrosis obliterated the walls of medium-sized veins in ethmoturbinates. Severe ulceration and desquamation of the main olfactory epithelium (MOE) manifested in vomeronasal organ (VMO) and septum. Intense infiltrates of neutrophils surrounded and occasionally involved centrifugal afferent sensory branches of olfactory (CNI), ethmoidal, and maxillary nerves (Fig 3A). Multifocal inflammation extended to the submucosa of maxillary and ethmoid sinuses at 6 dpi. Abundant viral antigen was detected in the sloughed necrotic mucosal epithelium intermixed with numerous intact and degenerate neutrophils, macrophages and variable amounts of fibrin. Lingual fungiform and circumvallate papillae were surrounded by neutrophils, which clustered on necrotic gustatory buds. The tongue was mildly swollen due to dissecting edema and neutrophilic interstitial glossitis in all 3 deer mice at 3 dpi. Abundant viral antigen was present in the nasal passages spanning sustentacular, olfactory and basal cells of MOE in all 3 of the deer mice at 3 dpi (Fig 3B) and less prominently in lingual mucosa, hard palate and oropharynx. Branches of chorda tympani, greater superficial petrosal and glossopharyngeal nerves (CNIX) were variably degenerate or minimally inflamed. Pterygopalatine (parasympathetic ganglion deep within pterygoid fossa in the upper jaw), petrosal and lateral geniculate nuclei (in tympanic bulla) and trigeminal ganglion were multifocally degenerate and surrounded and/or infiltrated by small numbers of neutrophils in 2 of the deer mice at 6 dpi. Virus antigen was present in the ganglia (trigeminal ganglion, Fig 3C) at 3 and 6 days of infection (S2 and S3 Figs). The glomerular layer of the main olfactory bulb (MOB) was spongiotic and immunoreactive at 3 dpi (Fig 3E). Laminar neuronal degeneration and scattered necrotic neurons were observed in anterior olfactory nucleus in the brain of one of the most affected deer mice at 6dpi. Histioneutrophilic inflammation manifested in frontal lobe of the brain of one of the deer mice by 6 dpi (Fig 3D and 3E). In inflamed areas of the MOB, viral antigen was also detected in cytoplasm of microglial and mitral cells (Fig 3F–3I). Viral antigen within the olfactory bulb (Fig 3J) remained prominent from 3 dpi to 6 dpi. In accordance with the olfactory bulb, detection of the virus was observed 3 dpi and 6 dpi in the nasal turbinates (Fig 3K). Interestingly, marked increase in SARS-CoV-2 antigen was observed at the 6 dpi timepoints within trigemimal nerve (Fig 3L), indicating that the virus was capable of invading nervous fibers in the subacute phase of the disease. This then allowed access to rostral most brain regions by the 6 dpi time-point resulting in glial phenotypic changes inducing microcytosis. Less severe glial reactions and immunoreactivity were present multifocally within brain stem at the level of lateral sulcus nucleus (NTS), optic chiasm, hypothalamus, and thalamic parabrachial nucleus (PbN), ventral posteromedial nucleus (VPM) culminating in gustatory cortex. Neuronal cell bodies in the affected regions of olfactory lobe, brain stem, thalamus, hypothalamus and insula showed variable immunoreactivity against viral nuclear capsid. Individual neurons were shrunken with angular borders, hypereosinophilic cytoplasm and pyknotic nuclei. Retinal ganglionic and inner nuclear layers showed multifocal immunoreactivity (S4–S6 Figs). There were no significant histologic lesions in peripheral or central nervous systems at 14 dpi. Calvarial bone marrow showed multifocal cytoplasmic immunoreactivity in myeloid precursors, including megakaryocytes, at 6 dpi (S7 Fig). Inflammation and mucosal desquamation were mild to moderate in trachea and main stem bronchi showed mild to moderate immunoreactivity, respectively, at 3 dpi. Immunoreactivity was also detected in mononuclear and stellate, antigen presenting cells in reactive tracheobronchial and hilar lymph nodes at 6 dpi. Extensive histioneutrophilic and hemorrhagic bronchointerstitial pneumonia manifested at 3- and 6-days dpi along with leukocytoclastic vasculitis involving main and medium sized branches of the pulmonary artery along with marked peribronchiolar and perivascular lymphoid hyperplasia (S8 Fig). Terminal bronchioles and adjacent alveoli contained infiltrates of moderate to large numbers of macrophages and neutrophils intermixed with multifocal extravasated erythrocytes. Abundant viral antigen was detected in individual bronchiolar lining epithelial cells and scattered alveolar lining cells. Some inflammatory foci were interspersed by multinucleate syncytial cells. Perivascular mild neutrophilic infiltrates were present around main blood vessels at the base of the heart and around renal hilar vessels and nerves at 6 dpi (S10 Fig). Other visceral organs did not show significant pathologies. Occasional multinucleate cells of epithelial and histiocytic origin were scattered amongst inflammatory infiltrates. Inflammation significantly subsided at 14 dpi where lungs showed mild pulmonary fibrosis and residual pneumonia. Hemorrhage was less prominent to absent in lungs of infected deer mice by 14 days. Lamina proprial cellularity of the small intestine, particularly duodenum and ileum, moderately increased by 6 dpi and immunoreactivity was detected in crypt epithelium and mature enterocytes along with occasional submucosal macrophages (S9 Fig).

### SARS-CoV-2 transmission

**Fig 4:**
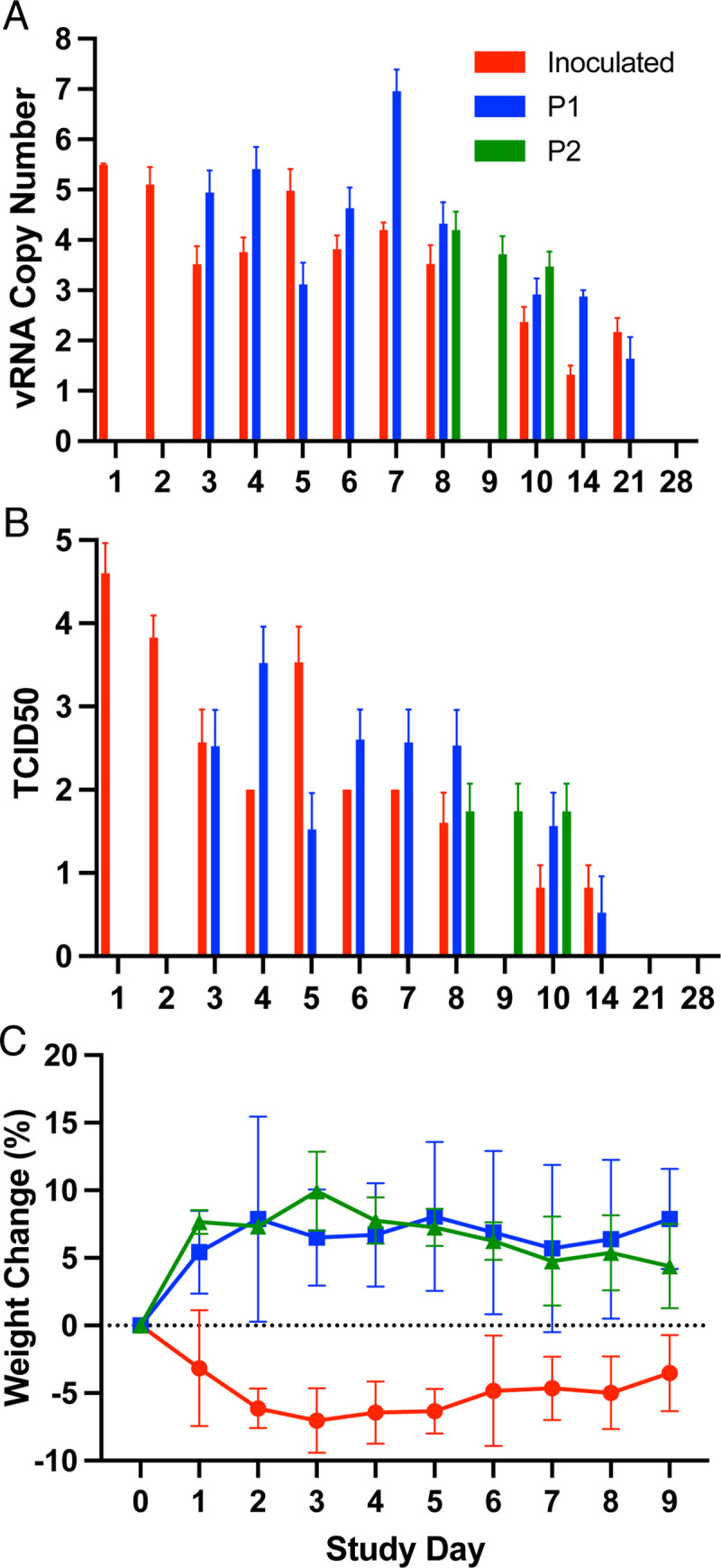
Abundance of viral RNA in oral swabs and weight loss in deer mice infected with SARS-CoV-2. (A) Probe-based PCR was used to detect levels of E gene RNA and quantified against an E gene plasmid standard (Integrated DNA Technologies). Transient weight loss occurred in some deer mice inoculated with SARS-CoV-2. (B) Virus isolation from oral swabs of infected deer mice. *P2 contact deer mice were euthanized on day 10 to prepare P2 virus stock. Deer mice were weighed each day during the transmission study (C). All three inoculated deer mice, one passage 1 deer mouse (DM6) and both passage 2 (DM8, DM9) deer mice lost weight followed by recovery.

To assess transmission, 3 deer mice were intranasally inoculated with 2x10^4^ TCID_50_ of virus and the next day they were moved to a new cage containing 3 naive contact deer mice. Oral swabs, but not rectal swabs, of the three contact deer mice (DM5, DM6, DM7) became vRNA^+^ on days 2, 5 and 5 post-contact (P1, days 3 and 6 of the study, Table 1). As they became vRNA^+^, the P1 deer mice were transferred to a third cage containing 2 additional naive contact deer mice (P2). The P2 deer mice (DM8, DM9) had detectable vRNA from oral swabs 5 days after contact (day 8 of the study), demonstrating that sustained natural transmission can occur. The inoculated and P1 deer mice were held until day 28 post contact, at which time all had antibodies detectable by western blot (S1 Fig). The P2 contact deer mice were euthanized on day 14 of the transmission study to provide a passage 2 viral stock. Viral RNA (Fig 4A) was detected up to 21 days post inoculation, whereas infectious virus (Fig 4B) was detected up to 14 days post inoculation. Each of the inoculated deer mice in the transmission study lost weight in the first 4–6 days before regaining weight, as did one passage 1 deer mouse (DM6) and both passage 2 deer mice (DM8, DM9) but to a lesser extent (Fig 4C).

**Table 1:** Detection of vRNA in oral swabs.

Study day	DM1	DM2	DM4	DM5	DM6	DM7	DM8	DM9
**1**	3.3E+05	3.2E+05	3.0E+05					
**2**	8.1E+04	3.0E+05	1.3E+03	Neg	Neg	Neg		
**3**	8.2E+03	1.3E+03	4.3E+02	2.7E+05	Neg	Neg		
**4**	4.4E+03	1.1E+03	1.2E+04	7.8E+05	Neg	Neg	Neg	Neg
**5**	2.8E+05	1.8E+03	4.6E+02	3.9E+03	Neg	Neg	Neg	Neg
**6**	3.2E+03	3.1E+03	1.3E+04	6.0E+03	1.2E+05	2.6E+03	Neg	Neg
**7**	2.0E+04	1.9E+04	7.9E+03	1.1E+05	2.7E+07	2.9E+03	Neg	Neg
**8**	1.1E+03	2.8E+02	8.6E+03	6.2E+04	1.4E+03	3.7E+02	7.7E+02	3.1E+04
**9**							3.7E+02	1.0E+04
**10**	4.7E+02	5.3E+00	2.3E+02	7.2E+02	1.8E+03	2.2E+00	9.0E+02	5.1E+03
**14**	1.3E+01	1.6E+01	3.3E+01	4.5E+02	9.0E+02	9.0E+02	Euth	Euth
**21**	Neg	1.9E+02	2.6E+02	Neg	Neg	1.3E+02		
**28**	Neg	Neg	Neg	Neg	Neg	Neg		
	Inoculated			Contact P1			Contact P2	

Values = vRNA copy numbers. Gray boxes, no sample collected. Neg, no vRNA detected by PCR. Euth, day of euthanasia of deer mice DM8 and DM9.

### Detection of spike protein insert

**Fig 5:**
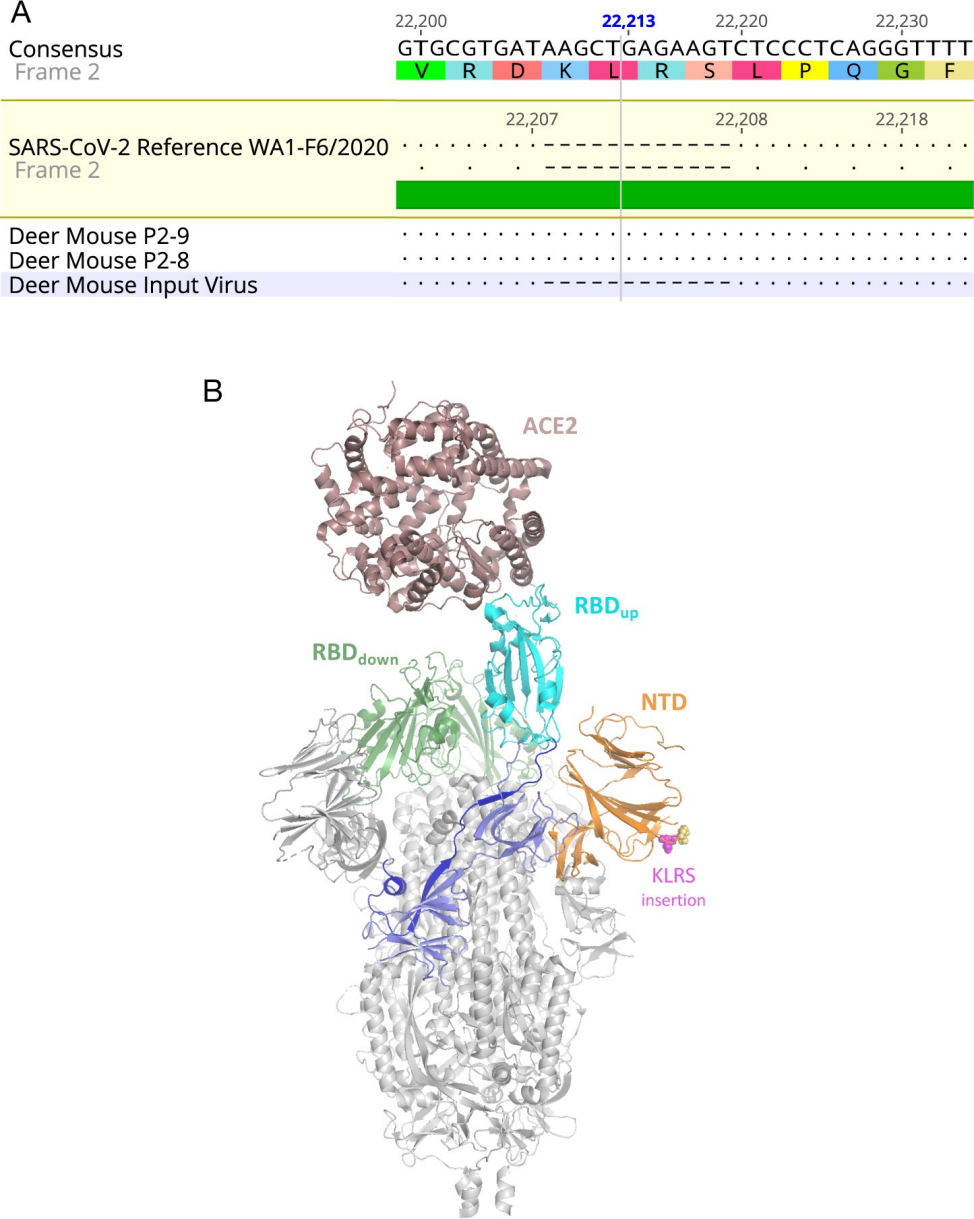
Spike protein insertion after serial passage in deer mice. (A) Passage of SARS-CoV-2 led to fixation of a 4-residue insertion in the N-terminal domain. Oral swabs of both DM8 and DM9 passage 2 deer mice (P2-8, P2-9, respectively) were submitted to RNA-Seq and an insertion was detected in all reads spanning residues 216–219 (nt 22,208–22,209, hatches) of the spike protein, implying a strong purifying selection for the insert during passage. (B) Homology model of the spike protein-human ACE2 complex showing the location of the KLRS insertion observed in the P2 virus. Model was generated by threading the deer mouse P2 spike protein sequence into the EM structure of the open-state SARS-CoV-2 spike protein (PDB: 6VYB) and colored as follows: Core (grey), N-terminal domain (NTD, orange) with KLRS insert as magenta spheres and native loop residues as yellow spheres, interdomain linker (blue), receptor binding domain (RBD, cyan for one up-conformation copy, green for two down-conformation copies), SD1/SD2 domains (light blue). The structure of the human ACE2 receptor (brown) was then superposed on the up-confirmation RBD using the crystal structure of the RBD-ACE2 complex (PDB: 6VW1).

The viral genome sequence was determined by NGS using oral swab samples from the two P2 deer mice and compared to the input virus sequence. A 4-residue insertion, KLRS, occurred in the N-terminal domain (NTD) of the spike protein, at residues 216–219 (nt 22,208–22,219) (Fig 5A) and was observed in all reads (150 reads for DM8, 140 reads for DM9). A structural model shows this insertion is located in a solvent-accessible loop that is distant from the RBD that interacts with ACE2 (Fig 5B). The surface location of the KLRS insert at a location away from the RBD may suggest interactions with an unknown co-receptor, and notably mouse coronavirus 1 (formerly mouse hepatitis virus) uses the NTD as a receptor binding domain [23]. It may be that the passage 2 deer mice were both infected by the passage 1 deer mouse DM5 (Table 1) that was introduced to the P2 cage when vRNA was initially detected in DM5 oral swabs 4 days prior to introduction of the other two passage 1 deer mice (DM6, DM7). However, the passage 2 deer mice did not become PCR^+^ until 2 days after DM6 and DM7 deer mice were added to the P2 cage, thus it cannot be conclusively determined which P1 deer mice transmitted the virus to the P2 deer mice.

Although the insertion was not detected in the NGS data for the input virus, which was sequenced at 8x coverage, conventional PCR using the forward primer containing the 12 nt insertion confirmed that the insert was present at low levels in the inoculum virus and at very low levels in the virus obtained from BEI Resources (S11 Fig). PCR with the insert primer also showed that all 17 infected deer mice in this study had at least some insert virus, implying a strong purifying selection during passage. In addition, two synonymous mutations occurred in the Orf7 (codon 5, CUU > CUA) and nucleocapsid (codon 145, CAC > CAU) genes in the P2 virus.

## Discussion

The order Rodentia has more than 2,200 species and the divergence of families Cricetidae (hamsters, voles, lemmings, and New World mice and rats) and Muridae (Old World mice, rats and gerbils) occurred about 18.5 mya [24]. Cricetidae has more than 600 species, including at least 70 species in the North American subfamily Neotominae [24] that include deer mice and other peromyscines. Few cricetid ACE2 sequences are available, but the 20 critical residues of ACE2 that interact with the RBD of SARS-CoV-2 [18] suggest other cricetid rodents are likely susceptible (S1 Table). In addition to *Peromyscus* species, two other neotomids from different genera have ACE2 similarities that suggest they are susceptible; the northern grasshopper mouse (*Onychomys torridus*) with 18/20 identities, and the desert woodrat (*Neotoma lepida*) with 17/20 identities. Experimental challenges of these species will be necessary to determine their susceptibility and which will shed light on diversity of North American species that may be susceptible to SARS-CoV-2. ACE2 residues are less human-like (15 of 20 residues) in the cricetid North American prairie vole (*Microtus ochrogaster*; subfamily Arvicolinae) and South American long-tailed pygmy rice rat (*Oligoryzomys longicaudatus*; subfamily Sigmodontinae), suggesting they are less likely to be susceptible. The laboratory house mouse (family Muridae) is not susceptible and it has 13 of the 20 critical ACE2 residues [18].

The respiratory pathology that occurred demonstrates that deer mice are a suitable small animal model for the study of SARS-CoV-2 disease. Neutralizing antibody was detected on day 6 post challenge, which corresponded with an inability to isolate infectious virus from lungs or olfactory bulbs, suggesting rapid antibody control of the virus. The ELISA, which is IgG-specific, detected high titer antibody on day 14, but not day 6, suggesting the neutralizing antibody on day 6 was exclusively IgM. No deer mouse anti-IgM detection antibodies are available, thus it is not possible to definitively demonstrate IgM reactivity. Despite this seroconversion and neutralization, we isolated virus from oral swabs of some deer mice to 14 days post infection.

Elevated inflammatory immune signatures by qPCR also suggested that a proinflammatory adaptive immune response may contribute to the pathogenesis of disease, which is also thought to be a significant component of COVID-19 morbidity and mortality [25]. The elevated expression of CD8β, Tbx21 transcription factor and IL-21, and low but significant expression of IL-2Rα, suggest CTL infiltration and activation [26]. We did not detect robust IFNγ or IL-6 expression, both of which are associated with fatal COVID-19 [22], which may account for why the deer mice did not have significant disease or death. Although gene expression is suggestive of biological responses, the inability to detect the proteins encoded by these genes in deer mice limits interpretation of gene expression results. New approaches for detection and quantification of immune proteins will be required to further scrutinize the host response in deer mice.

Neurological manifestations and neuropathogenesis of human coronaviruses have been dwarfed by the respiratory component of the diseases [27]. Understanding the underlying mechanisms of neuroinvasion was among the main goals of this study. Involvement of peripheral nerves of a 3-year-old girl spontaneously coinfected with HCoV 229E and OC43 resulted in acute flaccid paralysis (AFP), inability to masticate or swallow, speech loss and total lack of muscle tone and deep muscle reflexes [28]. In deer mice, the presence of virus in the peripheral cranial nerves, ganglia and the brain suggests a wide spectrum of neurological consequences that could precipitate parasympathetic and sympathetic clinical symptoms such as xerostomia, epiphora, trigeminal neuralgia, confusion, and more consistently hyposmia/anosmia and hypogeusia/ageusia [29,30]. Bidirectional trans-neuronal dissemination in the medulla can result in rapid death if the more vital neighboring respiratory center becomes infected. SARS-CoV-2/deer mouse infection studies may clarify the complex relationship between sensory losses, chronic pain and compromise to the BBB and immune system of affected COVID-19 patients. If cervical ganglia are infected, autonomic dysfunction, and potential myogenic effect in addition to brain stem injury could lead to global brain ischemia due to electrographic myocardial injury and constriction of cervical and skull arterial blood supply [31]. Collective pathology and immunohistochemistry results point to two different mechanisms that SARS-CoV-2 may utilize to enter the central nervous system. Similar to herpesviruses, early in the course of the disease at 3 dpi, the virus appeared to invade the brain in a retrograde axonal transmission along gustatory, olfactory and trigeminal pathways, bypassing the BBB [32]. Penetration routes can occur through damaged olfactory epithelium or via infected monocytes through compromised BBB later in the course of the disease. The neuropathology induced in this animal model makes it more suitable to further elaborate on neuropathogenesis of COVID-19 when compared to the Syrian hamster model which develop marked respiratory disease [33].

Deer mice are among the most widely studied rodents in North America and are frequently collected by mammalogists conducting field work. Outbreaks of SARS-CoV-2 on farms in several U. S. states [34] has resulted in the deaths of thousands of American mink (*Neovison vison*) and escape of other infected mink [35]. Many of these farms are located in deer mouse habitat, thus the risk of transmission to deer mice that inhabit mink barns or escape of SARS-CoV-2-infected mink, could lead to spillback to rodents. There is now evidence that wild mink in Utah have become infected with SARS-CoV-2, likely via contact with caged or escaped mink [36]. This heightens concerns that deer mice or other neotomine rodents could serve as secondary reservoirs of SARS-CoV-2 in North America. Dromedary camels, which are secondary reservoirs of MERS-CoV, had detectable vRNA in oral swab samples for up to 35 days in an experimental infection study [37]. All contact deer mice in this study became infected with detection of vRNA in oral swabs as early as 2 days after contact, and inoculated deer mice had detectable vRNA to 21 days, suggesting that sustained transmission among wild deer mice is possible. The susceptibility of two cricetid rodents, deer mice and Syrian hamsters, also raises the possibility that cricetid rodents could have served as intermediate hosts in spillover to humans and/or recombination events between coronaviruses. As a precedent for recombination, alphacoronaviruses have been detected in cricetid rodents in Europe, including bank voles (*Myodes glareolus*) and field voles (*Microtus agrestis*), and in those studies each of the alphacoronaviruses possessed spike genes derived from betacoronaviruses [38]. Considering that cricetid rodents are also found in Asia, it must be considered that they could have served as intermediate hosts for SARS-CoV-2 prior to spillover into humans [5].

The weight loss of the three inoculated deer mice could be reflective of a higher dose inoculum, or greater virulence of the wildtype virus that was abundant in the inoculum relative to the NTD insertion virus and will require further investigation. No SARS-CoV-2 sequences in Genbank have this insertion, thus it may not have relevance to human infection. It remains unclear whether this insertion rose in frequency to near fixation due to bottlenecks during transmission and/or systemic spread of the virus, or to positive selection. Ongoing studies will address this issue.

Deer mice and other peromyscine rodents have been used in biomedical research for many decades, including for their roles as reservoir hosts of zoonotic agents [19,39,40], and aging and diabetes studies [41], both of which are comorbidities associated with higher mortality rates in humans [42]. They can live eight years in captivity, about four times longer than laboratory mice and Syrian hamsters, making them particularly suitable as a model organism to examine the effects of age and SARS-CoV-2 infection, and the durability of immunity to infection and vaccination. Moreover, as an outbred model, deer mice are more likely to reflect the diverse outcomes of infection observed in humans (e.g., neuropathology), in contrast to those that occur in highly inbred laboratory mice and Syrian hamsters [43].

We have examined SARS-CoV-2 infection in Syrian hamsters using the same virus stock and dose described in this study [44]. While both Syrian hamsters and deer mice show bronchointerstitial pneumonia that is thought to be a significant reason for the severe COVID-19 in some human patients, total lung area affected in hamsters seems to be larger, precipitating respiratory clinical signs. The elements of pulmonary inflammation in both species seem to be the same including early necrotizing and later proliferative beronchiolitis, leukocytoclastic vasculitis and proliferative pneumonia with formation of syncytial cells. Lethality of the respiratory disease in both experimental models is lacking, but in deer mice the expression of IFNγ and IL-6 does not markedly change in the course of acute and subacute phases of the disease, which may in part explain why the infected deer mice recovered without an overt or severe respiratory disease. At 6 dpi, lymphocytic infiltrates around respiratory ducts and blood vessels in this study seemed to intensify in deer mice, which is concordant with increased expression of the T cell genes (Fig 2) and a decline in viral replication (Fig 1).

Although no manifest behavioral signs were observed in the current study, SARS-CoV-2 infectious virus was detected in the brains of infected deer mice. Presence of infectious virus in the autonomic nervous system of the deer mice that survived the acute infection is in line with the COVID-19 complications seen in some human patients, especially the sympathetic and parasympathetic long-term complications. Infection of the CNS is not indicative of a lethal outcome in most human patients. Infection of autonomic nervous of Syrian hamsters and autonomic instability is less documented when compared to the deer mice used in the current model.

Collectively, the present study offers a novel animal model for the study of SARS-CoV-2 pathogenesis that has many similarities to the Syrian hamster, but also some differences. The availability of alternative models may facilitate efforts to understand tissue and organ specific pathogenesis to identify commonalities and differences that may shed light on mechanisms of SARS-CoV-2 pathogenesis.

## Methods

### Ethics statement

This study was approved by the Colorado State University Institutional Animal Care and Use Committee (approval number 993).

### Virus and cells

SARS-CoV-2 (isolate 2019-nCoV/USA-WA1, NR52281) was provided by BEI Resources. Virus was passaged twice on Vero E6 cells (ATCC CRL-1586) in 2% FBS-DMEM containing 10,000 IU/ml penicillin and streptomycin at 37°C under 5% CO_2_ to generate stock virus used in these experiments. Virus was stored at -80°C. All work with infectious virus was performed at BSL-3 with approval from the Colorado State University Institutional Biosafety Committee.

### Animal procedures

Deer mice (*Peromyscus maniculatus nebrascensis*) of both sexes and of 6 months of age were kindly provided by Dr. Ann Hawkinson at the University of Northern Colorado. This colony was established with deer mice captured near Whitewater, CO in 2000 [45].

Deer mice were intranasally inoculated under inhalation isoflurane anesthesia with 2x10^4^ TCID_50_ SARS-CoV-2. On days 3, 6 and 14, three inoculated deer mice were euthanized, and one sham-inoculated deer mouse was euthanized on day 3 and the other two on day 14. Necropsies were performed for tissue RNA, virus isolation, and histopathology and immunohistochemistry.

To assess contact transmission, three deer mice were inoculated as described above. The next day, the deer mice were added to a cage with three naive contact deer mice (passage 1). Oral swabs were collected daily to determine infection status via qPCR. As the contact deer mice became PCR^+^ they were moved into another cage containing two additional naive contact deer mice (passage 2).

### Virus detection

Swabs in virus transport medium were vortexed thoroughly and centrifuged to pellet cellular debris. RNA was extracted from swab supernatant using the QiaAmp Viral RNA kit (Qiagen) according to the manufacturer’s instructions. Tissues were homogenized and RNA extracted using RNeasy Mini kit (Qiagen) following manufacturer instructions. For detection of viral RNA, a one-step real-time RT-PCR E gene assay was employed using the GoTaq 1-Step RT-qPCR kit (Promega). The Berlin E gene primer/probe/plasmid standard (IDT Technologies) kit was used to quantify viral copy numbers [46]. For virus isolation from lungs, the lower third of left lungs were homogenized in 500 μl of 10% FBS-DMEM with a stainless steel bead and a TissueLyser II (Qiagen). Samples were diluted 10^−3^ and inoculated on Vero E6 cells and scored for CPE 4 days later.

### Serological analysis

Truncated SARS-CoV-2 nucleocapsid (N) protein (AA133-416) was produced to reduce non-specific binding during antibody production [47–49]. A bacteria-codon optimized gBlock for the truncated protein was produced by IDT DNA Technologies and cloned into a pET28a bacterial expression with a C-terminal 6xHis tag using the NEBuilder Assembly Kit (New England Biolabs). Recombinant protein was expressed in BL21(DE3) pLysS *E*. *coli* and purified by nickel affinity and size exclusion chromatography essentially as previously described [50], with the exception of the use of 50 mM HEPES buffer (pH 7.4) and 500 mM NaCl throughout purification to reduce aggregation. Purity and identity of purified nucleocapsid protein was determined by SDS-PAGE and mass spectrometry at the CSU Proteomics and Metabolomics Core, respectively.

ELISA was performed by coating plates with 200 ng/100 μl recombinant N protein diluted in PBS (pH 7.2) overnight, then washed 3x with PBS. Plates were blocked with SuperBlock T20 (Thermo Scientific) for 30 minutes and washed. Heat inactivated serum samples (60°C 60 min) were diluted 1:100 in PBS, then serially diluted 2-fold and incubated with antigen for 1 hr at room temperature then washed 3x PBS-0.25% TWEEN-20/3x PBS. Goat anti-*Peromyscus leucopus* IgG(H&L)-HRP conjugate (SeraCare) at 1:1,000 was incubated for 1 hour followed by PBS-TWEEN-20/PBS washing. ABTS substrate (Thermo Fisher) was added and after 15 min absorbance (405 nm) recorded. The titers we determined by the reciprocal of the greatest dilution that was 0.200 OD above the mean of the negative control serum samples.

For western blot detection, infected Vero cell supernatants were subject to centrifugal filter concentration with molecular weight cutoff of 300 kDa (Pall Microsep 300K Omega). Enriched virus was inactivated with 2% sodium dodecyl sulfate (SDS) and protein concentration determined with a Pierce BCA protein assay kit (Thermo Scientific) according to manufacturer’s instructions. Eight micrograms of protein per lane were separated by 12% SDS polyacrylamide gel electrophoresis and blotted onto Immobilon-P nylon membranes (Millipore). After transfer, the blots were sectioned by lane, blocked, and individual lanes incubated with 1:100 dilutions of the deer mouse sera or with house mouse anti-nucleocapsid monoclonal antibody overnight. Primary antibodies from deer mice were detected with goat anti-*P*. *leucopus* IgG-HRP and developed with the TMB membrane peroxidase substrate system (3,3’,5,5’-tetramethylbenzidine, KPL). Images were scanned with a Visioneer One Touch 9420 scanner at a gamma value of 1.0, and all contrast adjustments were uniformly applied using Adobe Photoshop.

Serum neutralization was performed starting at 1:10 dilution with 2-fold dilution series. An equal volume of SARS-CoV (100 TCID_50_) was added (final dilution of 1:20) and incubated for 1 hr at 37°C. The mixture was plated on Vero E6 cells and scored for CPE after 6 days. The titer was reciprocal of the greatest dilution that conferred 100% protection.

### Immune gene expression profiling

Deer mouse immune gene expression profiling has been previously described [21]. Briefly, primers (S2 Table) for various immune genes were used to amplify cDNA collected from lungs using QuantiNova reverse transcription and SYBR Green I PCR kits (Qiagen). The ΔΔCq method was used with normalization within sample on GAPDH (ΔCq) and fold-change calculated for each gene against the means of the 3 sham inoculated control deer mice (ΔΔCq). Statistical assessment was performed using Kruskal-Wallis test (ANOVA) with Prism software (GraphPad, Inc.)

### Pathology

Three deer mice were humanely euthanized at 3-, 6- and 14 dpi and after retrieval of lungs and spleens, the remaining deer mice carcasses were fixed in 10% neutral buffered formalin (10% NBF) after opening abdominal and thoracic cavities to allow for gross inspection and formalin penetration. Fixed specimens were transferred after at least 3 days from BSL-3 facility to the Colorado State University Diagnostic Laboratories, BSL-2 for trimming. Skulls were bisected (hemi skulls) and decalcified in semiconductor grade formic acid and EDTA (Calfor, Cancer Diagnostics, USA) for 2–3 days. Oral cavity, salivary glands, olfactory bulb, cerebrum, cerebellum, and brain stem were thoroughly inspected for gross lesions. Decalcified skulls and visceral organs were processed, embedded in paraffin wax and 4–5 μm sections were stained with hematoxylin and eosin for blinded evaluation by the pathologist using Nikon i80 microscope (Nikon Microscopy).

### Immunohistochemistry

Sections from hemi skulls and visceral organs were stained using ultraView universal alkaline phosphatase red detection kit. Heat-induced epitope retrieval was performed on a Leica Bond-III IHC automated stainer using Bond Epitope Retrieval solution for 20 minutes. Viral nucleocapsid antigen was detected with a purified rabbit polyclonal antibody. Labeling was performed on an automated staining platform. Fast Red was used as chromogen and slides were counterstained with hematoxylin. Immunoreactions were visualized by a single pathologist in a blinded fashion. In all cases, normal and reactive mouse brain sections incubated with primary antibodies was used as a positive immunohistochemical control. Negative controls were incubated in diluent consisting of Tris-buffered saline with carrier protein and homologous nonimmune sera. All sequential steps of the immunostaining procedure were performed on negative controls following incubation.

### Immunofluorescence staining and tissue imaging

Paraffin embedded tissue sections were stained for SARS-CoV-2 nucleocapsid protein (1:500), microtubule-associated protein 2 (Abcam; ab32454; 1:500), ionized calcium binding adaptor molecule 1 (Abcam; ab5076; 1:50)/glial fibrillary acidic protein (Sigma; 3893;1:500) using a Leica Bond RXm automated staining instrument following permeabilization using 0.01% Triton X diluted in Tris-buffered saline (TBS). Blocking was performed with 1% donkey serum diluted in TBS. Sections were stained for DAPI (Sigma) and mounted on glass coverslips in ProLong Gold Antifade mounting medium and stored at ambient temperature until imaging. Images were captured using an Olympus BX63 fluorescence microscope equipped with a motorized stage and Hamamatsu ORCA-flash 4.0 LT CCD camera. Images were collected and regions of interest quantified with Olympus cellSens software (v 1.18) using an Olympus X-line apochromat 10X (0.40 N.A.), 20X (0.8 N.A.) or 40X (0.95 N.A.) air objectives, or Uplan Flour X100 oil immersion (1.3 N.A.) objective.

### Next-generation sequencing library preparation for positive NP samples

Viral RNA from positive deer mouse samples was prepared for Next-generation sequencing. Briefly, cDNA was generated using SuperScript IV Reverse Transcriptase enzyme (Invitrogen) with random hexamers. PCR amplification was performed using ARTIC network V3 tiled amplicon primers in two separate reactions by Q5 High-fidelity polymerase (NEB). First-round PCR products were purified using Ampure XP beads (Beckman Coulter). Libraries were prepared using the Nextera XT Library Preparation Kit (Illumina) according to manufacturer protocol. Unique Nextera XT i7 and i5 indexes for each sample were incorporated for dual indexed libraries. Indexed libraries were again purified using Ampure XP bead (Beckman Coulter). Final libraries were pooled and analyzed for size distribution using the Agilent High Sensitivity D1000 Screen Tape on the Agilent Tapestation 2200, final quantification was performed using the NEBNext Library Quant Kit for Illumina (NEB) according to manufacture protocol. Libraries were then sequenced on the Illumina MiSeq V2 using 2 x 250 paired end reads.

### Deep sequencing analysis

Next-generation sequencing data were processed to generate consensus sequences for each viral sample. MiSeq reads were demultiplexed, quality checked by FASTQC, paired-end reads were processed to removed Illumina primers and quality trimmed with Cutadapt, duplicate reads were removed. Remaining reads were aligned to SARS-CoV-2 reference sequence by Bowtie2 (GenBank: MT020881.1). Alignments were further processed, and quality checked, using Geneious software, consensus sequences were determined and any gaps in sequences were filled in with the reference sequence. Consensus sequences were aligned in Geneious. Based upon the insertion that was identified in the passage 2 deer mice, a PCR assay was developed to detect the presence of the insert using a forward primer (5’-AGTGCGTGATAAGCTGAGAAGT-3’; 12 nt insert sequence underlined) and reverse primer (5’-TAACCCACATAATAAGCTGCAGC) that generated a 183 bp product. A control forward primer (5’-GCACACGCCTATTAATTTAGTGC-3’) for detection of both wild-type (201 bp) or insert (213 bp) vRNA was designed 5’ to the insert primer.

### Model

The Phyre2 structure modeling engine [51] was used in the One-to-One Threading expert mode to generate a model of the deer mouse passage spike protein with the KLRS insert using the C chain of PDB entry 6VYB. The resulting model showed the insert is located at a surface loop on the N-terminal domain composed of residues 216–219 and predicts that this loop is enlarged to accommodate the 4-residue insertion. To illustrate the location of this insertion relative to the ACE2 receptor (Fig 4C), the structure of human ACE2 bound to the spike receptor binding domain (RBD), PDB entry 6VW1, was superposed on the up-conformation RBD from the B chain of the spike trimer [52,53].

## Supporting information

S1 Fig Serum response to SARS-CoV-2 structural proteins by infected deer mice. Protein from virus-enriched supernatants from SARS-CoV-2-infected Vero E6 cells, 8 μg protein per lane, were separated by SDS gel electrophoresis, and individual lanes from western blots were probed with 1:100 dilutions of sera from groups of three mock and infected deer mouse sera at times post infection as indicated and from contact controls at 28 dpi post exposure to infected animals. Prepared monoclonal antibody to the nucleocapsid protein served as control (N). Asterisks indicate predicted and known positions of structural proteins based on molecular weight: spike protein (S), cleaved spike 1 (S1), nucleocapsid (N), cleaved receptor binding domain (RBD), membrane (M), and envelope (E).
(TIF)

S2 Fig Mouse olfactory (1, 4) and trigeminal pathways (2,3). The three major sensory branches of trigeminal nerve: ophthalmic (V1), maxillary, mainly formed by union between posterior nasal and rhinopalatine nerves (V2) and thickest branch, mandibular nerve (V3). Viral spread occurs via sympathetic and/or parasympathetic fibers into corresponding pterygopalatine and trigeminal ganglia (2 and 3, respectively). SARS-CoV-2 dissemination may be facilitated by transmission of disrupted olfactory epithelium and the olfactory neurons (1) to finally infect the main olfactory bulb (4) at 6 dpi as detailed below. Meningeal nerve was also inflamed at that time [54].
(TIF)

S3 Fig Necrosis of circumvallate and foliate papillae at posterior tongue, 3 dpi (A) with close-up view of neutrophils clustering around a taste bud (arrowheads) (B). Dissecting edema dispersing lingual skeletal muscles apart, 3 dpi (C). Interstitial suppurative glossitis extending into branches of chorda tympani nerve (arrowhead) at anterior tongue, 3 dpi (D). Multifocal viral immunostaining is showing lingual mucosa and submucosa with occasional immunoreactivity in a hypertrophied neuronal cell body in one of chorda tympani nerve branches, 6 dpi (E). Close-up view of the hypertrophied neuron in lingual parenchyma (F, arrowhead). Geniculate nucleus in tympanic bulla showing strong immunoreactivity in ganglionic neurons (cell bodies) of greater superficial petrosal and chorda tympani nerve fibers, 6 dpi (control negative inset). Petrosal ganglion with attached CN IX shows strong immunoreactivity in corresponding ganglionic neurons 6 dpi. Strong immunoreactivity is seen in scattered neurons and glial cells mainly microglia in the area of the nucleus of the solitary tract (NST) (I). Bars = 100 μm.
(TIF)

S4 Fig Schematic illustration depicting gustatory nerves carrying taste information from the soft palate and tongue to brain stem of the deer mouse. Chorda tympani nerve on anterior tongue (red) (1) along with greater superficial petrosal nerve (green) (2) located on the soft palate conduct taste information from taste buds to cell bodies in the geniculate ganglion located within tympanic bulla. The glossopharyngeal nerve (CN IX, blue) conducts taste information from the circumvallate and posterior foliate papillae on posterior tongue to petrosal ganglion on the medial aspect of tympanic bulla. Information from taste ganglia is then transmitted into the nucleus tractus solitarius (NTS) (3) in medulla oblongata. In the medulla, taste responses are processed and then carried through parabrachial nucleus (PbN) and thalamic ventral posteromedial nucleus (VPM) to the primary gustatory nucleus in the insula. Perception of flavors is integrated with other avenues of sensation, particularly olfaction (8, 9). Temporary to persistent loss of taste is the one of the most consistent and common clinical symptoms reported in patients suffering from the mild form of COVID-19 (10). Pathologies of different components of the mouse gustatory system are following in S3A–S3I Fig.
(TIF)

S5 Fig Massive necrosis and ulceration of MOE with expansion of submucosa by fibrinosuppurative exudate, 3 dpi (A). Close-up of embedded nerve branches, which appear rarefied by extensive status spongiosus and minimal infiltration of neutrophils, 3 dpi (B). Maxillary sinuses from a control deer mouse show intact lining epithelium with no immunoreactivity (C), 3 dpi. Marked immunoreactivity is seen in lining and detached MOE, 3 dpi (D). Branches of facial nerve show moderate axonopathy (E, arrowhead). Pterygopalatine ganglion is multifocally cuffed by neutrophils with variable degeneration of the constituent neurons, 3 dpi (F). Ethmoidal nerves percolating cribriform plate showing multifocal suppurative neuritis and perineuritis (G, arrowhead). Severe congestion of meningeal vessels and rarefaction of meningeal nerve with histioneutrophilic perineuritis/meningitis, 3 dpi (H). Optic chiasm and hypothalamus show multifocal neuronal immunoreactivity, 6 dpi (I). Bars = 100 μm.
(TIF)

S6 Fig Retinal ganglionic cell bodies show multifocal immunoreactivity extending into inner plexiform layer (black arrowheads) and scattered bipolar cells in the inner nuclear layer (white arrowheads), 3 dpi. Bar = 100 μm.
(TIF)

S7 Fig Calvarium bone marrow show strong cytoplasmic immunoreactivity in myeloid precursors, including monocytes (upper arrowhead) and megakaryocytes (lower arrowhead), 6 dpi. Bar = 100 μm.
(TIF)

S8 Fig Grossly consolidated lung portions show massive infiltration of pulmonary parenchyma by numerous neutrophils and macrophages with peribronchiolar lymphoid hyperplasia (A). Main branches of pulmonary artery are infiltrated by neutrophils and cuffed by lymphoid follicles (B). Syncytial and histiocytic giant cells are dispersed among inflammatory cells expanding pulmonary interstitium and filling alveolar spaces (C). Lungs from a control mouse is within normal histologic limits with no immunoreactivity (D). Multifocal prominent bronchiolar and milder parenchymal immunostaining is seen in lungs 3 and 6 dpi (E). Mononuclear cells, macrophages, and antigen-presenting cells showing scattered cellular immunostaining in the paracortex (T-zone) area, 3 and 6 dpi (F). Bars = 100 μm.
(TIF)

S9 Fig Duodenum at 3 dpi shows mild focal histioneutrophilic duodenitis (A). Duodenum and ileum show moderate histioneutrophilic and lymphoplasmacytic enteritis (B and C). Small intestine from negative control deer mouse shows minimal nonspecific staining in goblet cells lining intestinal villi (D). Small intestine at 3 dpi shows marked immunoreactivity in the cytoplasm and apical border of mature enterocytes, crypt cells and scattered submucosal mononuclear cells consistent with macrophages (E). Bars = 100 μm.
(TIF)

S10 Fig Heart at 6 dpi, left subvalvular endocardium and myocardium shows minimal lymphocytic interstitial myocarditis (A) and more significant atrial suppurative perivascular myocarditis in right atrium (B). Kidney hilus shows mild perivascular suppurative inflammation that encircles small branches of renal nerves (C). Bars = 100 μm.
(TIF)

S11 Fig Detection of spike protein insert sequence by PCR. Forward primers were designed to amplify the wildtype or insert spike sequences. WT and insert were detected in both the deer mouse inoculum virus (Vero E6 passage 2) and original sourced BEI Resources virus. The BEI Resources virus had low amplification at 30 cycles that became greater at 40 cycles.
(TIF)

S1 Table Critical ACE2 residues of cricetid rodents involved in SARS-CoV-2 spike binding. ACE2 sequences were downloaded from Genbank and the 20 residues important for SARS-CoV-2 binding to human ACE2 were compared to those of various species to determine potential receptor binding of SARS-CoV-2 to ACE2 of each species.
(XLSX)

S2 Table Primers used for SYBR Green qPCR gene expression profiling. Primers used for the amplification of deer mouse genes for the PCR expression array. Each primer is listed 5’ to 3’.
(XLSX)
